# Spontaneous Non-Sustained Ventricular Tachycardia and Premature Ventricular Contractions and Their Prognostic Relevance in Patients with Cancer in Routine Care

**DOI:** 10.3390/cancers13102303

**Published:** 2021-05-12

**Authors:** Annemarie Albrecht, Jan Porthun, Jan Eucker, Andrew J.S. Coats, Stephan von Haehling, Antonio Pezzutto, Mahir Karakas, Hanno Riess, Ulrich Keller, Ulf Landmesser, Wilhelm Haverkamp, Stefan D. Anker, Markus S. Anker

**Affiliations:** 1 Department of Cardiology (CVK), Charité Universitätsmedizin Berlin, 13353 Berlin, Germany; annemarie.albrecht@charite.de (A.A.); wilhelm.haverkamp@charite.de (W.H.); s.anker@cachexia.de (S.D.A.); 2 Berlin Institute of Health Center for Regenerative Therapies (BCRT), 13353 Berlin, Germany; 3 German Centre for Cardiovascular Research (DZHK) Partner Site Berlin, 13353 Berlin, Germany; Ulf.Landmesser@charite.de; 4 Department of Cardiology (CBF), Charité Universitätsmedizin Berlin, 12203 Berlin, Germany; 5 Department of Health Sciences in Gjøvik, Norwegian University of Science and Technology, Campus Gjøvik (NTNU-Gjøvik), 2815 Gjøvik, Norway; jan@porthun.eu; 6 Department of Hematology, Oncology and Cancer Immunology, Campus Benjamin Franklin, Charité University Medicine Berlin, 12203 Berlin, Germany; jan.eucker@charite.de (J.E.); Ulrich.Keller@charite.de (U.K.); 7 IRCCS, San Raffaele, 00163 Rome, Italy; andrewjscoats@gmail.com; 8 Department of Cardiology and Pneumology, Heart Center Göttingen, University of Göttingen Medical Center, Georg-August-University, 37073 Göttingen, Germany; stephan.von.haehling@med.uni-goettingen.de; 9 German Center for Cardiovascular Medicine (DZHK), Partner Site Göttingen, 37099 Göttingen, Germany; 10 Department of Hematology and Oncology, Benjamin Franklin Campus, Charité University of Medicine Berlin, 12203 Berlin, Germany; antonio.pezzutto@charite.de; 11 Berlin Institute for Health Charité and Max-Delbrück Center, Campus Buch, Building 42-53, Lindenberger Weg 80, 13125 Berlin, Germany; 12 Clinic for General and Interventional Cardiology, University Heart Center Hamburg, 20246 Hamburg, Germany; m.karakas@uke.de; 13 German Center for Cardiovascular Research (DZHK), Partner Site Hamburg/Kiel/Lübeck, 20246 Hamburg, Germany; 14 Department of Hematology and Oncology, Charité—Campus Virchow-Klinikum (CVK), 13353 Berlin, Germany; hanno.riess@charite.de; 15 Max-Delbrück-Center for Molecular Medicine, 13125 Berlin, Germany; 16 German Cancer Consortium (DKTK) & German Cancer Research Center (DKFZ), 69120 Heidelberg, Germany; 17 Berlin Institute of Health (BIH), 13353 Berlin, Germany

**Keywords:** ventricular arrhythmia, non-sustained ventricular tachycardia, ventricular premature contractions, cancer, survival

## Abstract

**Simple Summary:**

It is largely unknown how frequently cancer patients seen in routine care show ventricular arrhythmias during 24-h electrocardiograms. We have found that non–sustained ventricular tachycardia episodes of ≥3 and ≥4 beats duration were more frequent in cancer patients than controls. Non–sustained ventricular tachycardia with ≥4 beats and ≥20 premature ventricular contractions/day seen in routine 24-h electrocardiograms of patients with cancer carry prognostic relevance.

**Abstract:**

**Aims:** It is largely unknown whether cancer patients seen in routine care show ventricular arrhythmias in 24 h electrocardiograms (ECGs), and whether when they are detected they carry prognostic relevance. **Methods and Results:** We included 261 consecutive cancer patients that were referred to the department of cardiology for 24 h ECG examination and 35 healthy controls of similar age and sex in the analysis. To reduce selection bias, cancer patients with known left ventricular ejection fraction <45% were not included in the analysis. Non–sustained ventricular tachycardia (NSVT) episodes of either ≥3 and ≥4 beats duration were more frequent in cancer patients than controls (17% vs. 0%, *p* = 0.0008; 10% vs. 0%, *p* = 0.016). Premature ventricular contractions (PVCs)/24 h were not more frequent in cancer patients compared to controls (median (IQR), 26 (2–360) vs. 9 (1–43), *p* = 0.06; ≥20 PVCs 53% vs. 37%, *p* = 0.07). During follow-up, (up to 7.2 years, median 15 months) of the cancer patients, 158 (61%) died (1-/3-/5-year mortality rates: 45% [95%CI 39–51%], 66% [95%CI 59–73%], 73% [95%CI 64–82%]). Both non-sustained ventricular tachycardia of ≥4 beats and ≥20 PVCs/24 h independently predicted mortality in univariate and multivariate survival analyses, adjusted for all other univariate predictors of mortality as well as relevant clinical factors, including cancer stage and type, performance status (ECOG), prior potentially cardiotoxic anti-cancer drug therapy, coronary artery disease, potassium concentration, and haemoglobin (multivariate adjusted hazard ratios: NSVT ≥4 beats [HR 1.76, *p* = 0.022], ≥20 PVCs/24 h [HR 1.63, *p* < 0.0064]). **Conclusions:** NSVT ≥4 beats and ≥20 PVCs/day seen in routine 24 h ECGs of patients with cancer carry prognostic relevance.

## 1. Introduction

In retrospective cohort studies, cardiovascular (CV) death as cause of death is reported in about 10–30% of patients with cancer [1,2]. There are many anti–cancer therapies such as anthracylines, trastuzumab, and cyclophosphamide that are known to cause cardiotoxicity in up to half of cancer patients, depending on the cancer type and drug dosage [3]. In order to initiate preventive measures against cardiotoxicity in cancer patients there is an urgent need to find reliable biomarkers. With regards to blood biomarkers, troponin is helpful in monitoring cancer patients during anti–cancer therapy and in the identification of patients with an increased risk of cardiotoxicity [4]. Previous research has shown that the resting heart rate in cancer patients without significant cardiovascular disease is a significant and independent predictor of all-cause mortality in multivariate survival analysis [5].

We hypothesized that cancer patients in routine care experience metabolic changes—caused by anti-cancer therapy and the overall catabolic state of the cancer disease—that could result in significant ventricular arrhythmias associated with worse survival. Since ventricular arrhythmias are frequently seen in patients with reduced left ventricular ejection fraction (LVEF) [6], we excluded patients with known LVEF <45%. We know of no other study that has examined the overall occurrence of ventricular arrhythmia in cancer patients in any real world clinical setting. In this retrospective, observational cohort study we examined the presence of spontaneous non-sustained ventricular tachycardia (NSVT), the length of the NSVT runs, and the number of premature ventricular contractions (PVCs) detected during routine 24-h electrocardiogram (24 h ECG) recordings, as well as their prognostic relevance in routinely treated cancer patients.

## 2. Methods

### 2.1. Study Population

Between 1 February 2012, and 31 May 2018, the total number of adult (age ≥ 18 years) cancer patients cared for on the oncology wards of Campus CBF of the Charité was 39,699. Of these patients, we reviewed the medical records of all cancer patients with histologically confirmed cancer who had undergone a 24-h ECG recording (*n* = 277). These 277 24-h ECG recordings represent 1.4% of all 19,810 24-h ECG recorded in the Cardiology Department in the time frame of interest. None of these 277 cancer patients had a repeat 24-h ECG in the study period. Patients with known left ventricular ejection fraction (LVEF) <45% were excluded (*n* = 16)—leaving 261 patients with a cancer diagnosis and an available 24-h ECG for analyses, which represents a subgroup of 0.7% (261/39,699) of all cancer patients cared for in that time period in Campus CBF of Charité (median time between 24-h ECG and echocardiogram 1 day [interquartile range 0–10 days]).

In the patients of interest, we reviewed the medical records for cancer stage and entity, clinical and laboratory baseline characteristics, ECOG (Eastern Cooperative Oncology Group) performance status [7], echocardiograms, and secondary diagnoses, closest to the 24-h ECGG recording. We followed all cancer patients for all-cause mortality through 3 July 2019. Additionally, we included a retrospective cohort of 35 generally healthy controls that received a 24-h ECG, echocardiogram, and routine blood chemistry as part of a preventive examination in 2018. All control subjects had similar age and sex compared to the cancer patients, and were free of cancer and significant cardiovascular disease, other than controlled arterial hypertension.

All cancer patients were followed-up for all-cause mortality by monitoring of the electronic database of the Charité. The study plan was approved by the Charité ethics committee and complies with the Declaration of Helsinki.

### 2.2. 24-h Electrocardiograms

A standard 24-h ECG was performed in all patients. CardioDay Version 2.4.5 (Getemed AG, Teltow, Germany) was used for analysis of 24-h ECGs and the results were verified by AA, MSA, and WH. We recorded total number of premature atrial contractions and premature ventricular contractions (PVCs), presence and length of non-sustained ventricular tachycardia (NSVT), and average 24-h heart rate (in beats per minute, bpm). Following the recommendations of the WHO and EHRA/HRS/APHRS [8,9], NSVT was defined as ≥3 consecutive heartbeats exceeding a heart rate of 100 beats per minute and originating from the ventricular heart chamber.

### 2.3. Statistical Analyses

Data are presented as either mean ± standard deviation (SD) or median with interquartile range (IQR). The Kolmogorov–Smirnov test was used to assess normal distribution. We used unpaired Student’s *t*-test to compare two normally distributed groups and Mann–Whitney U test for non-normally distributed data. The contingency tables were preferably analysed with Chi-squared test. For 2 × 2 tables with at least one cell assignment smaller than five, we used the Barnard’s test [10,11,12]. For survival analyses, we used the Cox-proportional hazard survival analyses (log-rank test). Results are given as hazard ratios (HRs) with 95% confidence intervals (95% CI) for risk factors. Kaplan Meier curves were generated for illustration purposes. Since this study was designed to identify patterns, it was unnecessary to test for multiplicity and the significance tests are descriptive [13,14]. For statistical analyses, we used “R 1.1.463”, “Stat View 5.0 software” (SAS Institute, Inc., Cary, NC, USA), and “IBM SPSS Version 25.0” (IBM, Armonk, NY, USA). We considered a *p*-value < 0.05 as statistically significant in all analysis.

## 3. Results

### 3.1. Study Population

We included 261 consecutive cancer patients (129 with solid cancers and 132 patients with haematological malignancies, Table S1) that were referred to the department of cardiology for 24-h ECG examination, and 35 healthy controls of similar age and sex (all Caucasians). Baseline characteristics are shown in Table 1 and baseline medication in Table S2. In 181 cancer patients and all 35 controls, LVEF was assessed. LVEF was similar between cancer patients and controls (65 ± 7 vs. 64 ± 7, *p* = 0.81). Follow-up of patients ended on July 3, 2019, after a median of 15 months (maximum 7.2 years). 188 patients (72%) had an advanced cancer stage (≥III). 158 cancer patients (61%) died during follow-up (1-/3-/5-year mortality: 45% [95%CI 39–51%], 66% [59–73%], 73% [64–82%]).

199 patients (76%) had previously received some kind of anti-cancer drug therapy before and 162 patients (62%) some kind of potentially cardiotoxic anti-cancer drugs [3]. Haemoglobin, sodium, and potassium concertation were lower in cancer patients vs. controls (median time between 24-h ECG and blood sample 1 day [interquartile range 0–2 days]). Since only generally healthy controls were included, secondary diagnoses and different drug treatments were more frequently observed in cancer patients than in the controls (Table 1, Tables S2 and S3).

### 3.2. 24-h Electrocardiograms

**Figure 1:**
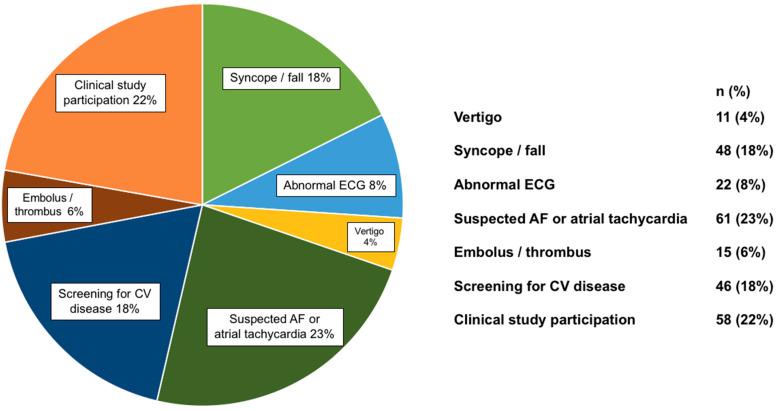
Indication for 24-h ECG in cancer patients (*n* = 261). ECG, Electrocardiogram; CV, cardiovascular event; AF, atrial fibrillation.

**Figure 2:**
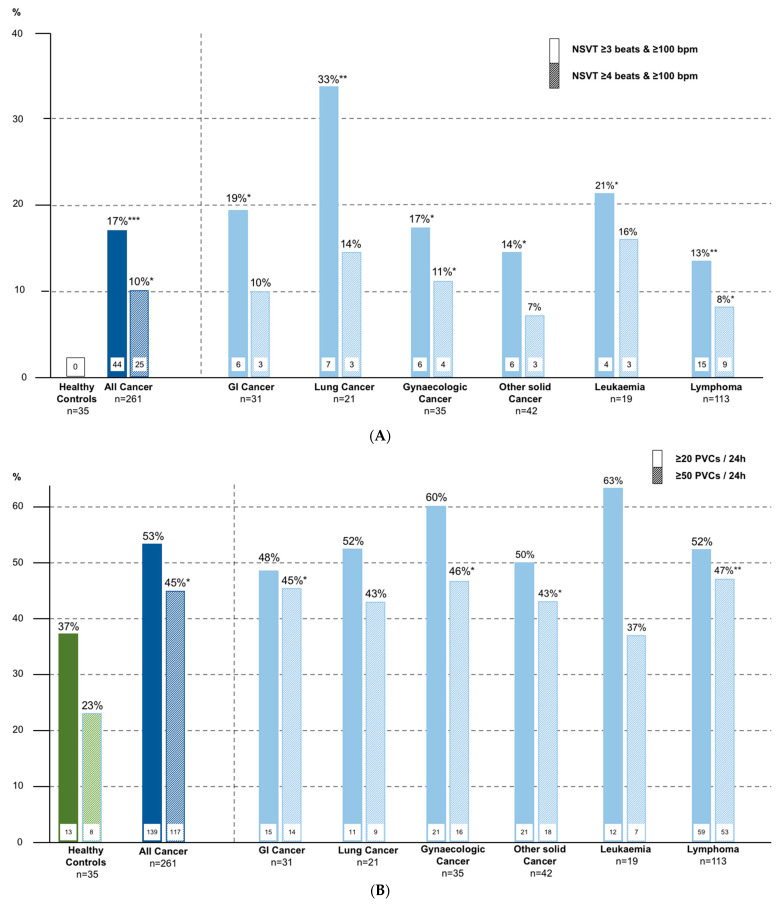
(**A**) Proportion of subjects with NSVT ≥3 beats & ≥4 beats. NSVT, non-sustained ventricular tachycardia; GI, gastrointestinal; bpm, beats per minute, * *p* < 0.05, ** *p* < 0.01, vs. healthy controls; (**B**) Proportion of subjects with ≥20 PVCs & ≥50 PVC/24 h. PVCs, premature ventricular contractions; GI, gastrointestinal; bpm, beats per minute, * *p* < 0.05, ** *p* < 0.01, *** *p* < 0.001, vs. healthy controls.

The most frequent reasons for recording a 24 h ECG were syncope or falls, suspected atrial fibrillation or atrial tachycardia, screening for CV disease, or clinical study participation (each ~20%, Figure 1). Analysis of all 24-h ECGs showed that non–sustained ventricular tachycardia (NSVT) with ≥3 and ≥4 beats were more frequent in cancer patients than in the controls (17% vs. 0%, *p* = 0.0008; 10% vs. 0%, *p* = 0.016)—Table 1, Figure 2A). Premature atrial contractions/24 h was not significantly increased in cancer patients vs. the controls. Premature ventricular contractions (PVCs)/24 h showed a tendency towards higher frequency in cancer patients (median (IQR), 26 (2–360) vs. 9 (1–43), *p* = 0.06; ≥20 PVCs 53% vs. 37%, *p* = 0.07; ≥50 PVCs 23% vs. 45%, *p* = 0.013 (Figure 2B). Average 24-h heart rate was higher in cancer patients. In cancer patients with NSVT ≥4 beats and ≥20 PVCs/24 h, LVEF was similar to patients without these arrhythmias (Tables S4 and S5).

### 3.3. Comparing Patients with and without Ventricular Arrhythmias

Subgroup analyses showed that patients with NSVT ≥4 beats more frequently had coronary artery disease, more often used angiotensin-converting enzyme inhibitors, had higher average 24 h heart rate, and more PVCs/24 h (Table S4). Patients with ≥20 PVCs were older, had higher average 24 h heart rate, had more premature atrial contractions/24 h and NSVT with ≥4 beats, were more often female, had more frequently arterial hypertension, coronary artery disease, and previous myocardial infarction, and more often used angiotensin-converting enzyme inhibitor (Table S5).

### 3.4. Comparing Patients with and without Death during Follow-Up

We compared patients with vs. without a fatal event during follow-up (Table 1). Patients with a fatal event had higher age, more frequently advanced cancer stage, and solid cancers, and lower body mass index (BMI), haemoglobin, and sodium levels. NSVT ≥4 beats were more frequent in patients with fatal event (13% vs. 4%, *p* = 0.0066). Patients with fatal events had a higher number of PVCs/24 h (median (IQR), 54 (4–467) vs. 12 (1–280), *p* = 0.013) and more frequently ≥20 PVCs/24 h (60% vs. 43%, *p* = 0.0059).

### 3.5. Survival Analyses

**Figure 3:**
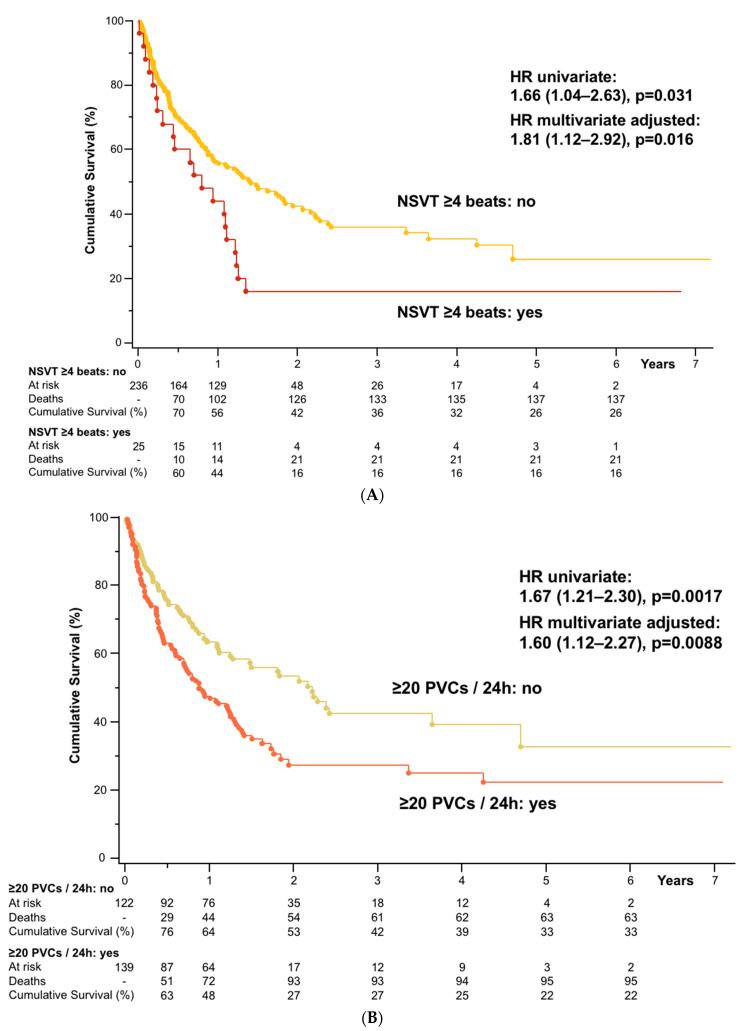
(**A**) Cumulative Survival of all cancer patients **(***n* = 261). NSVT, non-sustained ventricular tachycardia; (**B**) Cumulative Survival of all cancer patients (*n* = 261). PVCs, premature ventricular contractions.

Univariate and multivariate Cox-proportional hazard analyses in all cancer patients showed that NSVT ≥4 beats and ≥20 PVCs/24 h were associated with higher all-cause mortality (Table 2). Of note, NSVT ≥3 beats was not a significant predictor of mortality (univariate HR 1.22, 0.82–1.81, *p* = 0.32). For both risk factors (i.e., NSVT ≥4 beats and ≥20 PVCs/24 h), we conducted two multivariate models. In the first model, we included all univariate predictors of death and in the second additionally all other clinically relevant variables. Univariate Cox-proportional hazard analyses showed that PVCs/24 h in continuous analyses were no significant predictor of mortality. ROC analyses regarding the number of PVCs per day in cancer patients identified ≥20 PVCs as the best nominal predictor of mortality (area under the curve 59.1%, 95%CI 52.0%-66.3%), which was therefore used for the multivariate survival analysis described above. ROC analyses including the highest number of connected PVCs with a heart rate ≥100 bpm identified NSVTs with ≥4 beats as the best nominal predictor of mortality (area under the curve 57.4%, 95%CI 50.4%-64.4%), which was therefore used for the multivariate survival analysis described above. For illustrative purposes, we constructed Kaplan-Maier curves showing the survival benefit of patients without NSVT ≥4 beats or ≥20 PVCs (Figure 3A,B).

## 4. Discussion

This retrospective study shows that spontaneous NSVTs are seen more frequently in cancer patients in a clinical routine setting compared to what is found in healthy control subjects of similar age and sex. The number of PVCs/day also showed a tendency towards a higher incidence in cancer patients. The presence of NSVT of ≥4 beats and ≥20 PVCs/day independently carried relevant prognostic information.

It is important to note that this was a retrospective investigation, and we did not control the reasons for which these cancer patients underwent 24-h ECG recording, other than excluding patients with an LVEF <45%. There may have been selection biases with these cancer patients more likely having other CV risks than in an unselected cohort of cancer patients. It is of note, therefore, that we have recently published the results of a prospective study in 120 unselected patients with solid cancers without severe CV disease (diagnoses: colon, lung, or pancreatic cancer) who were followed for up to 12.5 years for survival [15]. In that study we found that both NSVTs (defined as ≥3 beats & heart rate ≥100 bpm), were present in 8% of cancer patients and the absolute number of PVCs per day predicted mortality (multivariate adjusted hazard ratio for NSVT [HR 2.44, *p* < 0.05] and PVCs [per 100, HR 1.021, *p* < 0.05]). The best cut-off for mortality prediction with respect to PVCs in patients with pancreatic and colorectal cancer was ≥50 PVCs/day (HR 2.30, *p* < 0.01), which was present in 26% of pancreatic and 18% of colorectal cancer patients.

In comparison, all 24-h ECGs in the current retrospective cohort were commissioned by a clinician and therefore were highly preselected. Only 0.7% (277/39,699) of patients with cancer that were treated in our hospital during the investigational period between 2012–2018 received a 24-h ECG and no patient received more than one 24-h ECG in this time period. Therefore, even though we excluded patients with known LVEF <45%, the current cohort had a much higher pre-test probability of finding ventricular arrhythmias. Consequently, NSVTs during 24-h ECGs in patients with cancer were observed more often in this retrospective cohort (i.e., in 17% of cancer patients), representing a 125% (16.86%/7.50%) higher incidence than in the prospectively studied cohort of cancer patients. Regarding the number of PVCs per day (here a median of 26), this represents a 550% higher value than in the prospective cohort of cancer patients [15]. It appears that only prospective studies can give realistic estimates for the presence of arrhythmias in cancer patients. Retrospective studies give values that are false high.

Still, both studies found an increased incidence of NSVTs in cancer patients compared to healthy controls (both *p* < 0.05) [15]. In healthy people in general, NSVTs are only rarely seen—an analysis from 22 phase 1 studies with a total of 1273 healthy volunteers that received 24-h ECGs found that NSVTs occurred in only 0.7% of patients (*n* = 9/1273, 0.7%) [16]—showing that both the 8% and 17% incidence of NSVTs in cancer patients in our reports are considerably higher than in healthy people. On the other hand, the number of premature atrial contractions/day was not significantly different here (median 195 vs. 82, *p* = 0.22). To better understand these issues, further prospective and mechanistic studies are needed to better understand the underlying pathomechanisms.

In this retrospective study we found that the best cut-offs for survival prediction with regards to NSVT was slightly higher (≥4 beats) than in the prospective cohort (≥3 beats). The current results were multivariate adjusted for many confounders including age, haemoglobin and potassium levels, BMI, cancer type and stage, ECOG, prior potentially cardiotoxic anti-cancer drugs, and coronary artery disease. NSVTs with ≥3 beats did not predict mortality in this cohort. The best cut-off for mortality prediction regarding PVCs/day was low in both cohorts: in the retrospective cohort at ≥20 PVCs/day and in the prospective cohort at ≥50 PVCs/day [15]. In both studies, this represented circa 0.5 PVCs per hour or circa 0.02–0.04% of all heart beats in one day.

Our results show, that in general 24-h ECGs should be performed more often in cancer patients and that they can identify those cancer patients with an increased risk of death. We think, especially those patients with palpitations, syncopal events, or multiple PVCs during resting ECG analysis, should be considered for referral for 24-h ECG recordings. Only if 24-h ECGs in cancer patients are performed more often in clinical studies and daily clinical routine can this lead to a better understanding of the underlying mechanisms. According to the current ESC guideline on ventricular arrhythmias “in hemodynamically relevant NSVT, amiodarone (300 mg i.v. bolus) should be considered” [17]. Of course, these guidelines were not written with cancer patients in mind and should be tested in cancer patients. Future studies should also evaluate long-term treatments for cancer patients that have shown NSVTs and/or increased number of PVCs during 24-h ECGs.

Considering the limitations of this study, first of all, this study was of a retrospective nature; many different cancer entities were included in the analysis. In total, 35 different cancer entities were included (129 patients with solid cancers and 132 with haematological malignancies). The many different cancer entities in this study might be considered as a weakness of this study, but at the same time, they could also be regarded as a strength of this study representing a real world scenario of all cancer patients that were assessed with 24-h ECGs at our hospital during six consecutive years.

Most of the patients had an advanced disease stage (72%) with a high mortality burden overall (median survival 15 months). Since the treating physicians had decided in all cancer patients that a 24-h ECG was indicated based on clinical judgement, it has to be acknowledged, that the patients in this cohort are a highly selected subgroup, and very likely represent a high risk group of cancer patients for CV problems, even though patients with a known LVEF <45% (6% of all cancer patients, LVEF was known in 197 of 277 cancer patients [71%]) were excluded. Therefore, the results of this analysis may be affected by the higher CV risk in our cancer patients and this may partly explain the high (17%) rates of NSVTs of ≥3 beats during 24-h ECG recordings. In absolute terms, the subgroup of lymphoma patients showed the lowest NSVT rates at 13% and lung cancer patients the highest at 33%. As we have shown before, in unselected CV risk cancer patients, only 8% of patients showed NSVTs with ≥3 beats [15]. Therefore, the true burden of NSVT in advanced cancer patients on average is most likely somewhere between 8–17%.

In this study we only analysed 24-h ECGs. None of our patients had longer ECG recordings, e.g., for 7–10 days. It has been shown before, that the median change of PVCs per day during 14-day ECG recordings is about 10% (IQR 5–15%) [18]—underlining the importance of longer ECG recordings. At the same time, ECG recordings in general are still rare in cancer patients in Germany. Future studies should focus on this.

We could not assess cause of death in any detail for all patients. Unfortunately, it is notoriously difficult to ascertain the cause of death in cancer patients. The main reason for this is because cancer patients often die at home or in hospices. Many cancer patients and families do not want an autopsy to be performed after death, since it would be of no benefit to them or the patient. Nonetheless, other much larger studies have found that, irrespective of the time after cancer diagnosis, 20–30% of cancer patients die due to CV disease [2,19,20], demonstrating that it is important to find new CV predictors of death in cancer patients.

We have previously shown that chemotherapy naïve cancer patients demonstrate mildly reduced LVEF [21]. Additionally, we have found that advanced cancer patients, irrespective of their prior treatment and other concomitant factors, on average have an elevated resting heart rate and that these cancer patients with heart rates ≥75 bpm have an increased mortality [5]. Subsequently, we have demonstrated in a large prospective cohort of 548 cancer patients that even in treatment naïve patients, an elevated resting heart rate is independently associated with increased mortality. We recently published the hypothesis that advanced cancer patients develop a new form of degenerative cardiomyopathy that was termed “cardiac wasting-associated cardiomyopathy” [22,23]. We think that it is caused by the many different factors that influence the function of the heart, including neurohormones, onco-metabolites, cytokines, oxidative and metabolic stress, hypoxia, fluid overload, chemo-, immune, and radiotherapy [21,22,23,24]. Together, all these factors can lead to cardiomyocyte dysfunction, apoptosis, fibrosis, ventricular remodeling, cardiac wasting, and even arrhythmias [22,23]. Most of the data underlining this hypothesis came from preclinical studies in animals. For example, Karlstaedt et al. demonstrated that the oncometabolite d-2-hydroxyglutarate impairs the contractile function of hearts in mice [25]. In rodents with colon adenocarcinoma, it was found that fractional shortening of the hearts was significantly reduced compared to animals without cancer, and electron microscopy identified a disrupted myocardial ultrastructure with increased fibrosis in these animals [26]. In a mouse model with Lewis lung cell carcinoma, cancer development was associated with cardiac remodelling and disturbance of the electrical conduction in the heart (reduced axon length and less dense core vesicles in the axons) [27]. In humans, very few reports have examined this question. Nonetheless, a detailed autopsy study, looking at 177 autopsies of cancer patients, found that those patients with cachexia before death had significantly lower heart weight (19% lower heart weight) [28].

## 5. Conclusions

In conclusion, we found that cancer patients with primarily advanced disease develop an increased vulnerability of the heart leading to ventricular arrhythmias, which are associated with increased mortality. Retrospective studies show higher frequencies of arrhythmias in cancer compared to prospective studies. We hypothesize that anti-arrhythmic therapies for cancer patients may help in selected clinical scenarios and should perhaps be tested in future trials.

## Figures and Tables

**Table 1:** Baseline characteristics.

Variable	Healthy Controls (*n* = 35)	Cancer Patients (*n* = 261)	*p*-Value	Cancer Deaths (*n* = 158)	Cancer Survivors (*n* = 103)	*p*-Value
Clinical characteristics						
Age (years)	68 ± 6 (IQR 62–73)	68 ± 12 (IQR 61–76)	0.86	69 ± 10 (IQR 62–75)	65 ± 17 (IQR 58–76)	0.016
Female sex, *n* (%)	19 (54)	132 (51)	0.68	76 (48)	56 (54)	0.32
BMI (kg/m^2^)	26 ± 4	24 ± 5	0.28	25 ± 5	26 ± 5	0.049
Cancer stage ≥III, *n* (%)	–	188 (72)	–	123 (78)	65 (63)	0.0095
Cancer type: solid, *n* (%)	–	129 (49)	–	93 (59)	36 (35)	0.0002
ECOG performance status ≥2, *n* (%)	–	111 (43)	–	75 (47)	36 (35)	0.046
Prior potentially cardiotoxic anti-cancer drugs, *n* (%)	–	162 (62)	–	104 (66)	58 (56)	0.12
Left ventricular ejection fraction (%)	64 ± 7	65 ± 7 (*n* = 181)	0.81	64 ± 8 (*n* = 100)	65 ± 7 (*n* = 81)	0.27
Laboratory parameters						
Haemoglobin (g/dL)	14.2 ± 1.3	10.8 ± 2.0	<0.0001	10.6 ± 1.9	11.1 ± 2.1	0.040
Leucocytes (/nL)	6.6 (5.5–7.6)	6.5 (4.3-9.6)	0.99	6.9 (4.4–9.9)	6.1 (4.3–9.2)	0.40
Platelets (/nL)	227 ± 46	225 ± 166	0.97	231 ± 195	217 ± 108	0.48
Sodium (mmol/L)	141 ± 2	139 ± 4	0.0002	138 ± 4	140 ± 3	0.0027
Potassium (mmol/L)	4.2 ± 0.4	3.9 ± 0.5	0.0004	3.9 ± 0.5	3.9 ± 0.5	0.91
Creatinine (mg/dL)	0.87 ± 0.19	1.00 ± 0.59	0.22	1.03 ± 0.68	0.94 ± 0.40	0.23
GOT (U/L)	26 (22–28)	26 (19-37) (*n* = 171)	0.75	27 (19–39) (*n* = 101)	25 (19–34) (*n* = 70)	0.34
Secondary diagnoses						
Arterial hypertension, *n* (%)	7 (20)	132 (51)	0.0007	79 (50)	53 (51)	0.82
Coronary artery disease, (%)	0	34 (13)	0.0040	22 (14)	12 (12)	0.59
Atrial fibrillation, *n* (%)	0	14 (5)	0.08	8 (5)	6 (6)	0.79
Previous myocardial infarction, *n* (%)	0	20 (8)	0.033	13 (8)	7 (7)	0.67
Diabetes mellitus type 2, *n* (%)	0	50 (19)	0.0003	31 (20)	19 (18)	0.81
Chronic kidney disease, *n* (%)	0	41 (16)	0.0014	28 (18)	13 (13)	0.27
Previous stroke, *n* (%)	0	27 (10)	0.011	17 (11)	10 (10)	0.79
Current use of antibiotics, *n* (%)	0	45 (17)	0.0007	33 (21)	12 (12)	0.054
24 h-ECG						
Average 24 h heart rate (bpm)	70 ± 9	78 ± 14	0.0007	79 ± 16	77 ± 11	0.41
No. of premature atrial contractions/24 h	82 (27–302)	195 (27–1167)	0.22	227 (32–1226)	146 (24–761)	0.20
No. of premature ventricular contractions/24 h	9 (1–43)	26 (2–360)	0.06	54 (4–467)	12 (1–280)	0.013
≥20 Premature ventricular contractions/24 h, *n* (%)	13 (37)	139 (53)	0.07	95 (60)	44 (43)	0.0059
≥50 Premature ventricular contractions/24 h, *n* (%)	8 (23)	117 (45)	0.013	80 (51)	37 (36)	0.020
NSVT with ≥3 beats & ≥100 bpm, *n* (%)	0	44 (17)	0.0008	31 (20)	13 (13)	0.14
NSVT with ≥4 beats & ≥100 bpm, *n* (%)	0	25 (10)	0.016	21 (13)	4 (4)	0.0066

Values are means ± SD, or n (%), or for abnormal distributed data median and Interquartile Range (IQR). Significant p-values (*p* < 0.05) are bold. 24 h, 24 hour; bpm, beats per minute; BMI, body mass index; ECOG, Eastern Cooperative Oncology Group; GOT, glutamic oxaloacetic transaminase; No. of premature atrial contractions/24 h, total number of premature atrial contractions recorded per 24 h; No. of premature ventricular contractions/24 h, total number of premature ventricular contractions recorded per 24 h; NSVT, non-sustained ventricular tachycardia; mmol/L, millimoles per litre; g/dL, grams per decilitre; kg/m^2^, kilogram per square meter; U/L, Units per litre;/nL, per nanolitre; ms, milliseconds.

**Table 2:** Univariate and Multivariate survival analyses in cancer patients (*n* = 261).

**Variable**	**Univariate Model**							
	**HR**	**95% CI**	**χ^2^**	***p*-Value**				
**Significant and Clinically Relevant Variables**								
NSVT ≥4 beats & ≥100 bpm (yes vs. no)	1.66	1.04–2.63	4.6	0.033				
≥20 Premature ventricular contractions/24 h (yes vs. no)	1.67	1.21–2.30	9.9	0.0017				
Age (per 1 year)	1.01	1.001–1.03	4.3	0.039				
BMI (per 1 kg/m^2^)	0.95	0.91–0.98	8.1	0.0043				
Cancer stage (≥III vs. I/II)	1.66	1.14–2.42	7.0	0.0082				
Cancer type (solid vs. haematologic)	2.09	1.52–2.87	20.3	<0.0001				
ECOG performance status (≥2 vs. 0/1)	1.58	1.15–2.17	8.2	0.0043				
Haemoglobin (per 1 g/dL)	0.91	0.84–0.99	5.3	0.021				
Sodium (per 1 mmol/L)	0.94	0.90–0.98	10.4	0.0013				
Opioids (yes vs. no)	1.67	1.13–2.47	6.7	0.0097				
Antidepressants (yes vs. no)	1.85	1.14–2.99	6.2	0.013				
Prior potentially cardiotoxic anti-cancer drugs (yes vs. no)	1.41	1.02–1.96	4.2	0.039				
Sex (female vs. male)	0.80	0.59–1.10	1.9	0.17				
Potassium (per 1 mmol/L)	1.07	0.78–1.48	0.2	0.66				
Current use of antibiotics (yes vs. no)	1.29	0.88–1.90	1.7	0.19				
Coronary artery disease (yes vs. no)	0.99	0.63–1.56	0.001	0.98				
**Variable**	**Multivariate Model 1**				**Multivariate Model 2**			
	**HR**	**95% CI**	**χ^2^**	***p*-Value**	**HR**	**95% CI**	**χ^2^**	***p*-Value**
**Multivariate Survival Analyses in Cancer Patients with NSVT ≥4 beats & ≥100 bpm**								
NSVT ≥4 beats & ≥100 bpm (yes vs. no)	1.82	1.13–2.94	6.0	0.015	1.76	1.09–2.84	5.3	0.022
Age (per 1 year)	1.02	1.01–1.04	8.4	0.0038	1.02	1.01–1.04	8.2	0.0041
Sodium (per 1 mmol/L)	0.96	0.92–1.002	3.5	0.061	0.96	0.92–1.003	3.4	0.07
Haemoglobin (per 1 g/dL)	0.92	0.84–1.0003	3.8	0.051	0.92	0.84–1.004	3.5	0.06
BMI (per 1 kg/m^2^)	0.96	0.93–0.996	4.7	0.031	0.96	0.92–0.998	4.2	0.041
Opioids (yes vs. no)	1.33	0.88–2.03	1.8	0.18	1.33	0.87–2.04	1.7	0.19
Antidepressants (yes vs. no)	1.50	0.91–2.47	2.6	0.11	1.60	0.97–2.64	3.4	0.07
Cancer stage (≥III vs. I/II)	1.50	1.01–2.21	4.1	0.043	1.52	1.03–2.24	4.4	0.037
Cancer type (solid vs. haematologic)	2.46	1.75–3.46	26.9	<0.0001	2.54	1.80–3.59	27.7	<0.0001
ECOG performance status (≥2 vs. 0/1)	1.23	0.87–1.73	1.4	0.24	1.40	0.97–2.01	3.3	0.07
Prior potentially cardiotoxic anti-cancer drugs (yes vs. no)	1.68	1.18–2.40	8.2	0.0041	1.70	1.19–2.43	8.4	0.0037
Sex (female vs. male)					0.68	0.48–0.95	5.1	0.024
Potassium (per 1 mmol/L)					1.15	0.83–1.59	0.7	0.40
Current use of antibiotics (yes vs. no)					1.33	0.88–1.99	1.8	0.18
Coronary artery disease (yes vs. no)					1.02	0.62–1.67	0.004	0.95
**Multivariate Survival Analyses in Cancer Patients with ≥20 Premature Ventricular Contractions**								
≥20 Premature ventricular contractions/24 h (yes vs. no)	1.71	1.22–2.40	9.7	0.0019	1.63	1.15–2.31	7.4	0.0064
Age (per 1 year)	1.01	0.9991–1.03	3.4	0.07	1.02	0.99995–1.03	3.8	0.05
Sodium (per 1 mmol/L)	0.97	0.92–1.008	2.6	0.11	0.96	0.92–1.008	2.6	0.11
Haemoglobin (per 1 g/dL)	0.91	0.83–0.989	4.9	0.027	0.91	0.83–0.991	4.7	0.031
BMI (per 1 kg/m^2^)	0.96	0.92–0.993	5.3	0.021	0.96	0.92–0.996	4.6	0.032
Opioids (yes vs. no)	1.21	0.80–1.84	0.84	0.36	1.22	0.79–1.86	0.8	0.37
Antidepressants (yes vs. no)	1.60	0.97–2.64	3.5	0.06	1.67	1.01–2.75	4.1	0.044
Cancer stage (≥III vs. I/II)	1.64	1.11–2.42	6.1	0.014	1.63	1.10–2.41	6.0	0.014
Cancer type (solid vs. haematologic)	2.44	1.74–3.43	26.5	<0.0001	2.49	1.77–3.52	27.0	<0.0001
ECOG performance status (≥2 vs. 0/1)	1.30	0.92–1.84	2.3	0.13	1.47	1.02–2.11	4.4	0.037
Prior potentially cardiotoxic anti-cancer drugs (yes vs. no)	1.53	1.07–2.17	5.5	0.019	1.54	1.08–2.20	5.6	0.018
Sex (female vs. male)					0.69	0.49–0.97	4.6	0.03
Potassium (per 1 mmol/L)					1.14	0.83–1.57	0.6	0.42
Current use of antibiotics (yes vs. no)					1.24	0.82–1.86	1.0	0.31
Coronary artery disease (yes vs. no)					0.91	0.55–1.50	0.1	0.71

Hazard ratios are presented for continuously or binomially distributed variables. Model 1 includes all significant univariate predictors of mortality. Model 2 includes all significant univariate predictors of mortality and other clinically important variables. Significant *p*-values (*p* < 0.05) are bold. 24 h, 24 hour; BMI, body mass index; ECOG, Eastern Cooperative Oncology Group; bpm, beats per minute; CI, Confidence interval. HR, hazard ratio; NSVT, non-sustained ventricular tachycardia; mmol/L, millimoles per litre; g/dL, grams per decilitre; kg/m^2^, kilogram per square meter; U/L.

## Data Availability

The data presented in this study are available upon request from the corresponding author. The data are not publicly available due to ethical and legal issues.

## Supplementary Materials

The following are available online at https://www.mdpi.com/article/10.3390/cancers13102303/s1, Table S1. Kind of Cancer Patients (*n* = 261). Table S2. Baseline Medication. Table S3. Prior Chemotherapy, Immunotherapy and Targeted Therapy. Table S4. Association of NSVT ≥4 Beats & ≥100 bpm with Relevant Variables in all Cancer Patients (n = 261). Table S5. Association of <20 PVC & ≥20 PVC with Relevant Variables in all Cancer Patients (n = 261).
