# Supplementary material for: Spontaneous Non-Sustained Ventricular Tachycardia and Premature Ventricular Contractions and Their Prognostic Relevance in Patients with Cancer in Routine Care
Source: Cancers (Basel). 2021 May 12;13(10):2303. doi: 10.3390/cancers13102303 (PMC8151948; doi:10.3390/cancers13102303)
Supplement: Supplementary file 1 [file cancers-13-02303-s001.zip › cancers-1135244-supplementary.pdf]

Supplementary Materials

Spontaneous Non-Sustained Ventricular Tachycardia and Premature Ventricular Contractions and Their Prognostic Relevance in Patients with Cancer in Routine Care

Annemarie Albrecht, Jan Porthun, Jan Eucker, Andrew J. S. Coats, Stephan von Haehling, Antonio Pezzutto, Mahir Karakas, Hanno Riess, Ulrich Keller, Ulf Landmesser, Wilhelm Haverkamp, Stefan D. Anker and Markus S. Anker

Table S1. Kind of Cancer Patients (*n* = 261).

**Group**	**Type of Cancer**	**No. of Patients (*n*=261) *n*, (%)**	**Solid Cancer or Haematological Malignancy**
Group 1—Gastrointestinal cancer (*n* = 31)	Rectal cancer	4 (2)	Solid cancer
	Colon cancer	12 (5)	Solid cancer
	Duodenal cancer	1 (<1)	Solid cancer
	Pancreatic cancer	2 (1)	Solid cancer
	Gastric cancer	4 (2)	Solid cancer
	Esophageal cancer	6 (2)	Solid cancer
	Cholangiocellular carcinoma	12 (5)	Solid cancer
Group 2—Lung cancer (*n* = 21)	Non-small-cell-lung-cancer	15 (6)	Solid cancer
	Small-cell-lung-cancer	6 (2)	Solid cancer
Group 3—Gynaecologic cancer (*n* = 35)	Cervical- / Ovarian- / Uterine cancer	12 (5)	Solid cancer
	Breast cancer	23 (9)	Solid cancer
Group 4—Other solid cancers (*n* = 42)	Tonsillar / Laryngeal / Oro- / Epi- / Hypopharyngeal carcinoma	15 (6)	Solid cancer
	Urothelial carcinoma	6 (2)	Solid cancer
	Kidney cell carcinoma	2 (1)	Solid cancer
	Prostate carcinoma	7 (3)	Solid cancer
	Sarcoma	4 (2)	Solid cancer
	Choroid melanoma	5 (2)	Solid cancer
	Thyroid cancer	1 (<1)	Solid cancer
	Thymus carcinoma	1 (<1)	Solid cancer
	Peritoneal Mesothelioma	1 (<1)	Solid cancer
Group 5—Leukaemia (*n* = 19)	Chronic myeloid leukemia	1 (<1)	Haematological malignancy
	Acute myeloid leukemia	11 (4)	Haematological malignancy
	Myeloproliferative neoplasia	3 (1)	Haematological malignancy
	Myelodysplastic syndromes	3 (1)	Haematological malignancy
	Acute lymphocytic leukemia	1 (<1)	Haematological malignancy
Group 6—Lymphoma (*n* = 113)	B-cell-Non-Hodgkin-lymphoma	82 (31)	Haematological malignancy
	T-cell-Non-Hodgkin-lymphoma	5 (2)	Haematological malignancy
	Hodgkin-lymphoma	10 (4)	Haematological malignancy
	Multiple myeloma	16 (6)	Haematological malignancy

Table S2. Baseline Medication.

Variable	Healthy Controls (*n* = 35)	Cancer Patients (*n* = 261)	*p*-Value	Cancer Deaths (*n* = 158)	Cancer Survivors (*n* = 103)	*p*-Value
Aspirin, *n* (%)	2 (6)	63 (24)	0.07	39 (25)	24 (23)	0.80
Angiotensin-converting enzyme inhibitor, *n* (%)	2 (6)	57 (22)	0.11	40 (25)	17 (17)	0.09
Calcium channel blocker, *n* (%)	0	37 (14)	**0.0025**	20 (13)	17 (17)	0.38
Angiotensin II receptor blocker, *n* (%)	0	36 (14)	**0.0028**	16 (10)	20 (19)	**0.033**
Beta-blocker, *n* (%)	5 (14)	117 (45)	**0.0001**	74 (47)	43 (42)	0.42
Spironolactone, *n* (%)	0	7 (3)	0.23	5 (3)	2 (2)	0.61
Diuretics, *n* (%)	0	71 (27)	**<0.0001**	45 (28)	26 (25)	0.57
Anticoagulants, *n* (%)	1 (3)	93 (36)	**0.0002**	61 (39)	32 (31)	0.21
Antidiabetics or Insulin, *n* (%)	0	28 (11)	**0.0099**	17 (11)	11 (11)	0.98
Proton pump inhibitors, *n* (%)	1 (3)	167 (64)	**<0.0001**	98 (62)	69 (67)	0.41
Opioids, *n* (%)	0	46 (18)	**0.0006**	32 (20)	14 (14)	0.17
Antidepressants, *n* (%)	0	23 (9)	**0.021**	19 (12)	4 (4)	**0.013**
Corticosteroids, *n* (%)	0	73 (28)	**<0.0001**	51 (32)	22 (21)	0.055
Prior chemotherapy, *n* (%)	–	199 (76)	–	127 (80)	72 (70)	0.052
Prior immunotherapy, *n* (%)	–	125 (48)	–	75 (47)	50 (49)	0.87
Alkylating agents, *n* (%)	–	113 (43)	–	64 (41)	49 (48)	0.26
Platins, *n* (%)	–	74 (28)	–	56 (35)	18 (17)	**0.0016**
Anti-metabolites, *n* (%)	–	98 (38)	–	64 (41)	34 (33)	0.22
Topoisomerase inhibitors, *n* (%)	–	47 (18)	–	28 (18)	19 (18)	0.88
Antracyclines, *n* (%)	–	79 (30)	–	45 (28)	34 (33)	0.44
Antimitotics, *n* (%)	–	60 (23)	–	30 (19)	30 (29)	0.06
Taxanes, *n* (%)	–	30 (11)	–	24 (15)	6 (6)	**0.020**
Histone deacetylase inhibitors, *n* (%)	–	1 (<1)	–	0	1 (1)	0.34
Antibodies/ Immunotherapy, *n* (%)	–	105 (40)	–	63 (40)	42 (41)	0.88
Thyrosin kinase inhibitors, *n* (%)	–	13 (5)	–	11 (7)	2 (2)	**0.043**
Other kinase inhibitors, *n* (%)	–	3 (1)	–	3 (2)	0	0.77
Proteasom inhibitors, *n* (%)	–	17 (7)	–	9 (6)	8 (8)	0.51
Other chemotherapeutic agents, *n* (%)	–	16 (6)	–	11 (7)	5 (5)	0.51

Significant p-values ($p < 0.05$) are bold.

Table S3. Prior Chemotherapy, Immunotherapy and Targeted Therapy.

Variables	All Cancer Patients $n = 261$	GI Cancer $n = 31$	Lung Cancer $n = 21$	Gynaecologic Cancer $n = 35$	Other solid Cancers $n = 42$	Leukaemia $n = 19$	Lymphoma $n = 113$
Alkylating agents, *n*	113	0	1	13	2	4	93
Cyclophosphamide, *n**	77	0	1	10	0	2	64
Ifosfamide, *n**	15	0	0	0	1	0	14
Melphalan, *n*	14	0	1	0	0	0	13
Temozolomide, *n*	1	0	0	0	1	0	0
Dacarbazine, *n*	6	0	0	0	0	0	6
Procarbazine, *n*	3	0	0	0	0	0	3
Carmustine, *n*	7	0	0	0	0	0	7
Chlorambucil, *n*	3	0	0	0	0	0	3
Thiotepa, *n*	3	0	0	0	0	0	3
Treosulfan, *n*	3	0	0	3	0	0	0
Busulfan, *n*	2	0	0	0	0	2	0
Bendamustine, *n*	24	0	0	0	0	0	24
Platins, *n*	74	18	16	13	15	1	11
Carboplatin, *n*	26	1	8	12	2	0	2
Cisplatin, *n**	40	4	7	4	13	1	11
Oxaliplatin, *n*	13	12	0	0	0	0	1
Anti-metabolites, *n*	98	22	8	8	9	10	41
High-dose Methotrexate, *n*	24	0	0	0	1	1	22
Fluorouracil, *n*	27	18	0	2	6	0	1
Pemetrexed, *n*	1	0	0	0	1	0	0
Tioguanine, *n*	1	0	1	0	0	0	0
Capecitabine, *n*	13	7	1	5	0	0	0
Gemcitabine, *n*	18	1	7	4	1	1	4
Fludarabine, *n*	7	0	0	0	0	4	3
Trifluridine/Tipiracil, *n*	1	1	0	0	0	0	0
Clofarabine, *n**	0	0	0	0	0	0	0
Cytarabine, *n*	32	0	1	0	0	9	22
Topoisomerase inhibitors, *n*	47	8	8	1	2	3	25
Irinotecan, *n*	8	8	0	0	0	0	0
Topotecan, *n*	3	0	1	1	0	0	1
Mitoxantrone, *n*	5	0	0	0	0	3	2
Pixantrone, *n*	6	0	0	0	0	0	6
Etoposide, *n*	29	1	8	0	2	0	18
Antracyclines, *n*	79	0	1	13	3	7	55
Doxorubicin / Adriamycin, *n**	70	0	1	9	3	3	54
Pegylated liposomal Doxorubicin, *n**	3	0	0	3	0	0	0
Daunorubicin, *n**	5	0	1	0	0	3	1
Epirubicin, *n**	5	0	0	4	1	0	0
Idarubicin, *n**	3	0	0	0	0	2	1
Antimitotics, *n*	60	0	1	2	1	2	54
Vincristine, *n*	54	0	0	0	1	2	51
Vinorelbine, *n*	3	0	1	1	0	0	1
Vinblastine, *n*	6	0	0	0	0	0	6
Taxanes, *n*	30	5	2	18	5	0	0
Docetaxel, *n**	11	4	0	3	4	0	0
Eribulin, *n*	3	0	0	3	0	0	0
Paclitaxel, *n**	19	1	2	15	1	0	0
Protein-bound paclitaxel, *n**	1	0	0	1	0	0	0
Histone deacetylase inhibitor, *n*	1	0	0	0	0	0	1
Belinostat, *n*	1	0	0	0	0	0	1
Antibodies/ Immunotherapy, *n*	105	5	6	9	10	1	74

Alemtuzumab, *n*	1	0	0	0	0	0	1
Bevacizumab, *n**	10	3	0	6	0	0	1
Brentuximab, *n*	7	0	0	0	0	0	7
Cetuximab, *n*	13	4	0	0	9	0	0
Daratumumab, *n*	1	0	0	0	0	0	1
Isatuximab, *n*	1	0	0	0	0	0	1
Nivolumab, *n*	6	0	4	0	1	0	1
Olaratumab, *n*	1	0	0	0	1	0	0
Panitumumab, *n*	1	1	0	0	0	0	0
Pertuzumab, *n**	1	0	0	1	0	0	0
Pembrolizumab, *n*	2	0	1	1	0	0	0
Rituximab, *n*	71	0	0	0	0	1	70
Tomuzotuximab, *n*	1	0	0	0	1	0	0
Ofatumumab, *n*	1	0	0	0	0	0	1
Trastuzumab, *n**	2	0	0	2	0	0	0
Programmed death-ligand 1-Antibody, *n*	1	0	1	0	0	0	0
Thyrosin kinase inhibitors, *n*	13	1	2	1	3	1	5
Afatinib, *n**	2	0	2	0	0	0	0
Axitinib, *n**	0	0	0	0	0	0	0
Ibrutinib, *n**	5	0	0	0	0	0	5
Imatinib, *n**	1	0	0	0	0	1	0
Osimertinib, *n**	2	0	2	0	0	0	0
Pazopanib, *n**	1	0	0	0	1	0	0
Sunitinib, *n**	1	0	0	0	1	0	0
Sorafenib, *n**	2	0	0	0	2	0	0
Nilotinib, *n**	1	0	0	0	0	1	0
Lapatinib, *n**	1	0	0	1	0	0	0
Regorafenib, *n**	1	1	0	0	0	0	0
Other Kinase inhibitors, *n*	3	0	0	1	0	0	2
Idelalisib, *n*	2	0	0	0	0	0	2
Palbociclib, *n*	0	0	0	0	0	0	0
Ribociclib, *n*	1	0	0	1	0	0	0
Proteasom inhibitors, *n*	17	0	1	0	0	0	16
Bortezomib, *n**	17	0	1	0	0	0	16
Ixazomib, *n*	1	0	0	0	0	0	1
Other chemotherapeutic agents, *n*	16	0	0	0	4	1	11
Tretinoin (ATRA), *n*	1	0	0	0	0	1	0
Everolimus, *n**	1	0	0	0	1	0	0
Temsirolimus, *n**	3	0	0	0	0	0	3
Mitomycin, *n*	4	0	0	0	3	0	1
Bleomycin, *n*	4	0	0	0	0	0	4
Venetoclax, *n*	1	0	0	0	0	0	1
Lenalidomide, *n*	2	0	0	0	0	0	2
Pomalidomide, *n*	1	0	0	0	0	0	1
Romidepsin, *n*	1	0	0	0	0	0	1
Azacitidine, *n*	1	0	0	0	0	1	0

GI, gastrointestinal. * anti-cancer drugs that may cause left ventricular dysfunction (according to the "2016 ESC Position Paper on cancer treatments and cardiovascular toxicity" – Zamorano et al. EHJ. 2016).

Table S4. Association of NSVT ≥4 Beats & ≥100 bpm with Relevant Variables in all Cancer Patients (n = 261).

Variable	No NSVT ≥4 Beats & ≥100 bpm (*n* = 236)	NSVT ≥4 Beats & ≥100 bpm (*n* = 25)	*p*-value
Clinical characteristics			
Age (year)	67 ± 12	65 ± 13	0.30
Female sex, *n* (%)	123 (52)	9 (36)	0.13
BMI (kg/m²)	25 ± 5	26 ± 7	0.26
Cancer stage ≥III, *n* (%)	166 (70)	22 (88)	0.33
Cancer type: solid, *n* (%)	116 (49)	13 (52)	0.74
ECOG performance status ≥2, *n* (%)	100 (42)	11 (44)	0.88
Prior potentially cardiotoxic anti-cancer drugs, *n* (%)	147 (62)	15 (60)	0.82
Left ventricular ejection fraction (%)	65 ± 7 (*n*=163)	63 ± 10 (*n*=18)	0.41
Laboratory parameters			
Haemoglobin (g/dL)	10.9 ± 2.0	10.4 ± 1.9	0.33
Leucocytes (/nL)	6.3 (4.4 – 9.7)	6.9 (3.9 – 9.5)	0.97
Platelets (/nL)	228 ± 170	204 ± 117	0.50
Sodium (mmol/L)	139 ± 4	138 ± 3	0.64
Potassium (mmol/L)	3.9 ± 0.5	3.9 ± 0.5	0.99
Creatinine (mg/dL)	0.98 ± 0.53	1.16 ± 1.01	0.15
GOT (U/L)	26 (19 – 36) (*n*=161)	33 (23 – 66) (*n*=10)	0.27
Secondary diagnoses			
Arterial hypertension, *n* (%)	119 (50)	13 (52)	0.88
Coronary artery disease, *n* (%)	27 (11)	7 (28)	**0.019**
Atrial fibrillation, *n* (%)	12 (5)	2 (8)	0.91
Previous myocardial infarction, *n* (%)	16 (7)	4 (16)	0.61
Diabetes mellitus type 2, *n* (%)	46 (19)	4 (16)	0.92
Chronic kidney disease, *n* (%)	38 (16)	3 (12)	0.89
Previous stroke, *n* (%)	26 (11)	1 (4)	0.52
Current use of antibiotics, *n* (%)	42 (18)	3 (12)	0.79
Medication at study entry			
Aspirin, *n* (%)	56 (24)	7 (28)	0.64
Angiotensin-converting enzyme inhibitor, *n* (%)	46 (19)	11 (44)	**0.0048**
Calcium channel blocker, *n* (%)	33 (14)	4 (16)	0.93
Angiotensin II receptor blocker, *n* (%)	33 (14)	3 (12)	0.93
Beta-blocker, *n* (%)	107 (45)	10 (40)	0.61
Spironolactone, *n* (%)	5 (2)	2 (8)	0.64
Diuretics, *n* (%)	66 (28)	5 (20)	0.39
Anticoagulants, *n* (%)	82 (35)	11 (44)	0.36
Antidiabetics or Insulin, *n* (%)	27 (11)	1 (4)	0.52
Proton pump inhibitors, *n* (%)	152 (64)	15 (60)	0.66
Opioids, *n* (%)	44 (19)	2 (8)	0.44
Antidepressants, *n* (%)	21 (9)	2 (8)	0.97
Corticosteroids, *n* (%)	69 (29)	4 (16)	0.52
Anti-cancer therapy			
Prior chemotherapy, *n* (%)	178 (75)	21 (84)	0.62
Prior immunotherapy, *n* (%)	116 (49)	9 (36)	0.21
Alkylating agents, *n* (%)	103 (44)	10 (40)	0.73
Platins, *n* (%)	67 (28)	7 (28)	0.97
Anti-metabolites, *n* (%)	90 (38)	8 (32)	0.55
Topoisomerase inhibitors, *n* (%)	42 (18)	5 (20)	0.79
Antracyclines, *n* (%)	72 (31)	7 (28)	0.80
Antimitotics, *n* (%)	55 (23)	5 (20)	0.71
Taxanes, *n* (%)	28 (12)	2 (8)	0.89
Histone deacetylase inhibitor, *n* (%)	1 (<1)	0	0.76
Antibodies/ Immunotherapy, *n* (%)	96 (41)	9 (36)	0.65
Thyrosin kinase inhibitors, *n* (%)	13 (6)	0	0.14
Other kinase inhibitors, *n* (%)	3 (1)	0	0.52
Proteasom inhibitor, *n* (%)	16 (7)	1 (4)	0.89
Other chemotherapeutic agents, *n* (%)	13 (6)	3 (12)	0.75
24h-ECG			
Average 24h heart rate (bpm)	78 ± 13	85 ± 20	**0.016**
No. of premature atrial contraction / 24h	185 (27 – 1028)	690 (51 – 2073)	0.09

No. of premature ventricular contraction / 24h	20 (2 – 279)	439 (114 – 3263)	**0.0001**
≥20 Premature ventricular contractions / 24h, *n* (%)	118 (50)	21 (84)	0.09
≥50 Premature ventricular contractions / 24h, *n* (%)	97 (41)	20 (80)	**0.0002**

Values are means ± SD or n (%). 24h, 24 hours; BMI, body mass index; ECOG, Eastern Cooperative Oncology Group; bpm, beats per minute; GOT, glutamic oxaloacetic transaminase; no., number; NSVT, non-sustained ventricular tachycardia. *P*-values are determined using the unpaired *t*-test.

Table S5. Association of <20 PVC & ≥20 PVC with Relevant Variables in all Cancer Patients (*n* = 261).

Variable	<20 PVC (*n* = 122)	≥20 PVC (*n* = 139)	*p*-Value
Clinical characteristics			
Age (year)	65 ± 13	70 ± 11	**0.0018**
Female sex, *n* (%)	73 (60)	59 (42)	**0.0051**
BMI (kg/m^2)	21 ± 5	25 ± 5	0.94
Cancer stage ≥III, *n* (%)	93 (76)	95 (68)	0.16
Cancer type: solid, *n* (%)	61 (50)	68 (49)	0.86
ECOG performance status ≥2, *n* (%)	56 (46)	55 (40)	0.30
Prior potentially cardiotoxic anti-cancer drugs, *n* (%)	71 (58)	91 (65)	0.23
Left ventricular ejection fraction (%)	65 ± 7 (*n*=89)	64 ± 8 (*n*=92)	0.38
Laboratory parameters			
Haemoglobin (g/dL)	10.9 ± 2.1	10.7 ± 2.0	0.52
Leucocytes (/nL)	6.1 (4.3 – 9.1)	6.8 (4.4–9.6)	0.36
Platelets (/nL)	218 ± 121	232 ± 197	0.52
Sodium (mmol/L)	139 ± 4	139 ± 4	0.86
Potassium (mmol/L)	3.9 ± 0.5	4.0 ± 0.6	0.12
Creatinine (mg/dL)	1.01 ± 0.66	0.99 ± 0.52	0.83
GOT (U/L)	24 (18–36) (*n*=83)	28 (20–37) (*n*=88)	0.12
Secondary diagnoses			
Arterial hypertension, *n* (%)	55 (45)	87 (63)	**0.0046**
Coronary artery disease, *n* (%)	8 (7)	26 (19)	**0.0036**
Atrial fibrillation, *n* (%)	4 (3)	10 (7)	0.17
Previous myocardial infarction, *n* (%)	4 (3)	16 (12)	**0.010**
Diabetes mellitus type 2, *n* (%)	22 (18)	28 (20)	0.67
Chronic kidney disease, *n* (%)	15 (12)	26 (19)	0.16
Previous stroke, *n* (%)	13 (11)	14 (10)	0.88
Current use of antibiotics, *n* (%)	19 (16)	26 (19)	0.50
Medication at study entry			
Aspirin, *n* (%)	27 (22)	36 (26)	0.48
Angiotensin-converting enzyme inhibitor, *n* (%)	20 (16)	37 (27)	**0.046**
Calcium channel blocker, *n* (%)	18 (15)	19 (14)	0.80
Angiotensin II receptor blocker, *n* (%)	12 (10)	24 (17)	0.08
Beta-blocker, *n* (%)	53 (43)	64 (46)	0.67
Spironolactone, *n* (%)	3 (2)	4 (3)	0.85
Diuretics, *n* (%)	32 (26)	39 (28)	0.74
Anticoagulants, *n* (%)	39 (32)	54 (39)	0.25
Antidiabetics or Insulin, *n* (%)	17 (14)	11 (8)	0.12
Proton pump inhibitors, *n* (%)	80 (66)	87 (63)	0.62
Opioids, *n* (%)	19 (16)	27 (19)	0.42
Antidepressants, *n* (%)	13 (11)	10 (7)	0.32
Corticosteroids, *n* (%)	34 (28)	39 (28)	0.97
Anti-cancer therapy			
Prior chemotherapy, *n* (%)	87 (71)	112 (81)	0.08
Prior immunotherapy, *n* (%)	59 (48)	66 (47)	0.89
Alkylating agents, *n* (%)	48 (39)	65 (47)	**0.021**
Platins, *n* (%)	37 (30)	37 (27)	0.56
Anti-metabolites, *n* (%)	47 (39)	51 (37)	0.56
Topoisomerase inhibitors, *n* (%)	26 (21)	21 (15)	0.38
Antracyclines, *n* (%)	35 (29)	44 (32)	0.60
Antimitotics, *n* (%)	28 (23)	32 (23)	0.31
Taxanes, *n* (%)	12 (10)	18 (13)	0.39
Histone deacetylase inhibitor, *n* (%)	0	1 (1)	0.46
Antibodies/ Immunotherapy, *n* (%)	53 (43)	52 (37)	0.27

Thyrosin kinase inhibitors, *n* (%)	9 (7)	4 (3)	0.12
Other kinase inhibitors, *n* (%)	3 (2)	0	0.09
Proteasom inhobitors, n (%)	6 (5)	11 (8)	0.71
Other chemotherapeutic agents, *n* (%)	6 (5)	10 (7)	0.95
24h-ECG			
Average 24h heart rate (bpm)	77 ± 15	80 ± 13	**0.042**
No. of premature atrial contraction / 24h	53 (20–353)	543 (100–2287)	**<0.0001**
No. of premature ventricular contraction / 24h	2 (0–7)	297 (87–1312)	**<0.0001**
NSVT ≥4 beats & ≥100 bpm, *n* (%)	4 (3)	21 (15)	**<0.0001**
NSVT ≥6 beats & ≥100 bpm, *n* (%)	2 (2)	10 (7)	**0.028**

Values are means ± SD or n (%). 24h, 24 hours; BMI, body mass index; ECOG, Eastern Cooperative Oncology Group; bpm, beats per minute; GOT, glutamic oxaloacetic transaminase; no., number; NSVT, non-sustained ventricular tachycardia. Significant p-values ($p < 0.05$) are bold.
